# Supplementary material for: Half-Heusler alloys as emerging high power density thermoelectric cooling materials
Source: Nat Commun. 2023 Jun 6;14:3300. doi: 10.1038/s41467-023-38446-0 (PMC10244423; doi:10.1038/s41467-023-38446-0)
Supplement: Supplementary file 1 — Supplementary Information [file 41467_2023_38446_MOESM1_ESM.docx]

**Supplementary Materials**

**Half-Heusler Alloys as Emerging High Power Density Thermoelectric Cooling Materials**

Hangtian Zhu^1, 2, †,^ *, Wenjie Li^1, †,^ *, Amin Nozariasbmarz^1^, Na Liu^1^, Yu Zhang^1^, Shashank Priya^1,^ *, Bed Poudel^1,^ *

*1: Department of Materials Science and Engineering, Pennsylvania State University, University Park, PA 16802, USA*

*2: Current address: Beijing National Laboratory for Condensed Matter Physics, Institute of Physics, Chinese Academy of Sciences, Beijing 100190, China*

*: Corresponding Authors: H. Z. (htzhu@iphy.ac.cn), W.L. (wzl175@psu.edu), B. P. (bup346@psu.edu), and S. P. (sup103@psu.edu)

†: These authors contribute equally.


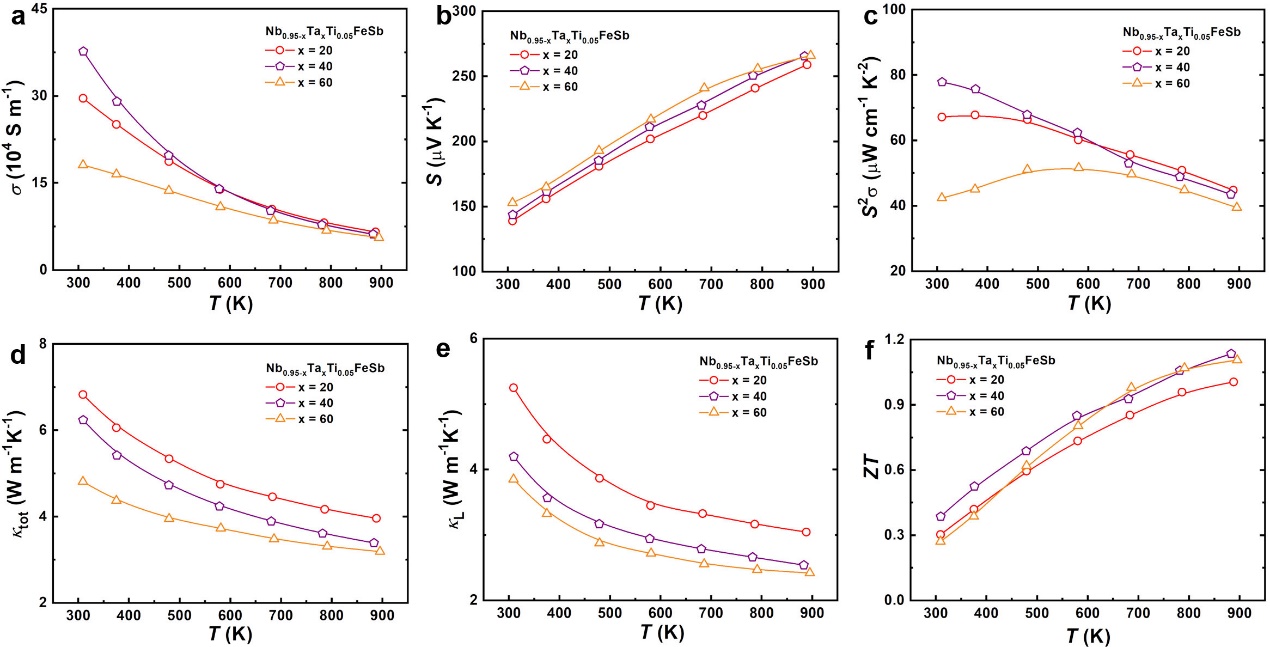


**Fig. S1. The thermoelectric transport properties of Ta alloyed Nb_0.95-x_Ta_x_Ti_0.05_FeSb samples after Sb-pressure control annealing at 1143 K for 2 days.** **a,** The temperature dependent electrical conductivity (*σ*). **b,** Seebeck coefficient (*S*). **c,** Power factor (*S^2^σ*). **d,** Total thermal conductivity (*κ_tot_*). **e,** Lattice thermal conductivity (*κ_L_*). **f,** *zT* value.


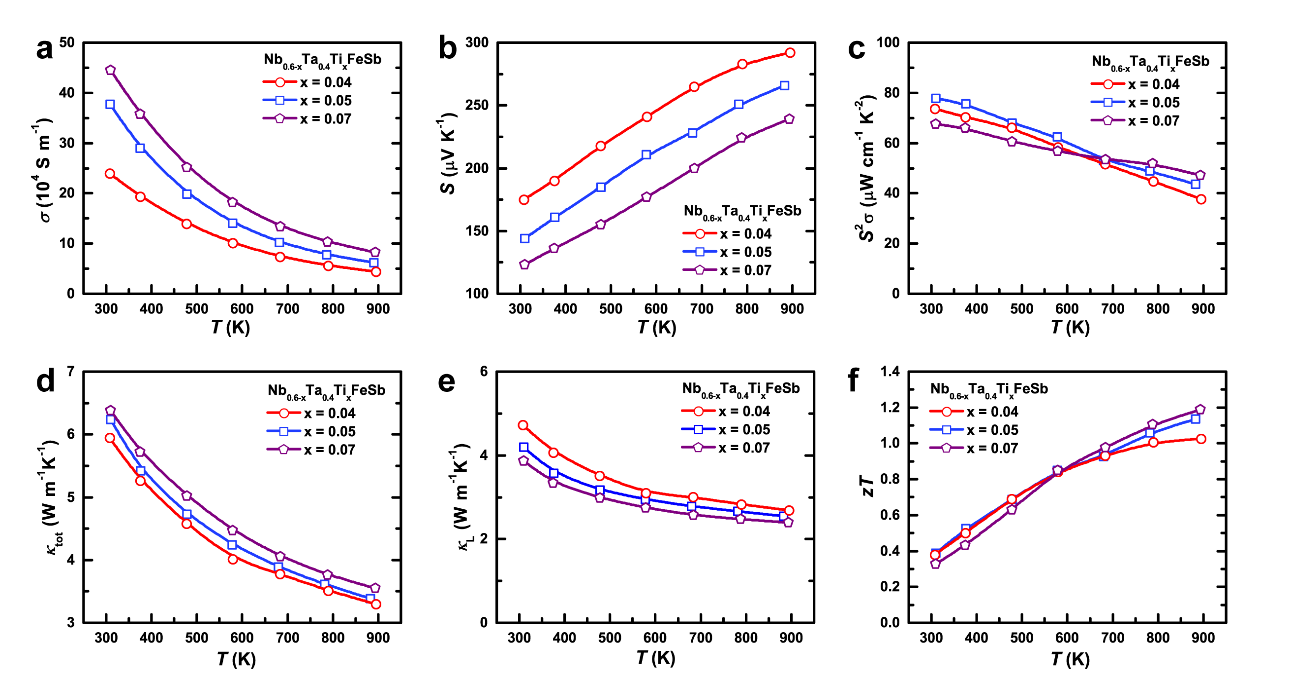


**Fig. S2 The thermoelectric performance of Ti doped Nb_0.60-x_Ta_0.40_Ti_x_FeSb samples after Sb-pressure control annealing at 1143 K for 2 days.** **a,** The temperature dependent electrical conductivity (*σ*). **b,** Seebeck coefficient (*S*). **c,** Power factor (*S^2^σ*). **d,** Total thermal conductivity (*κ_tot_*). **e,** Lattice thermal conductivity (*κ_L_*). **f,** *zT* value.


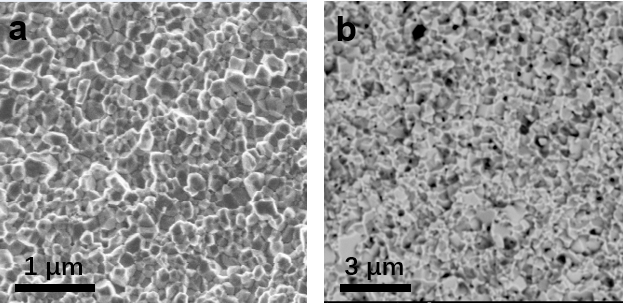


**Fig. S3 The SEM images of the Nb_0.55_Ta_0.40_Ti_0.05_FeSb samples.** **a,** The sample as-hot-pressed. **b,** After conventional vacuum annealing at 1123 K for 6 days. The gain size remains 200-300 nm afterwards.


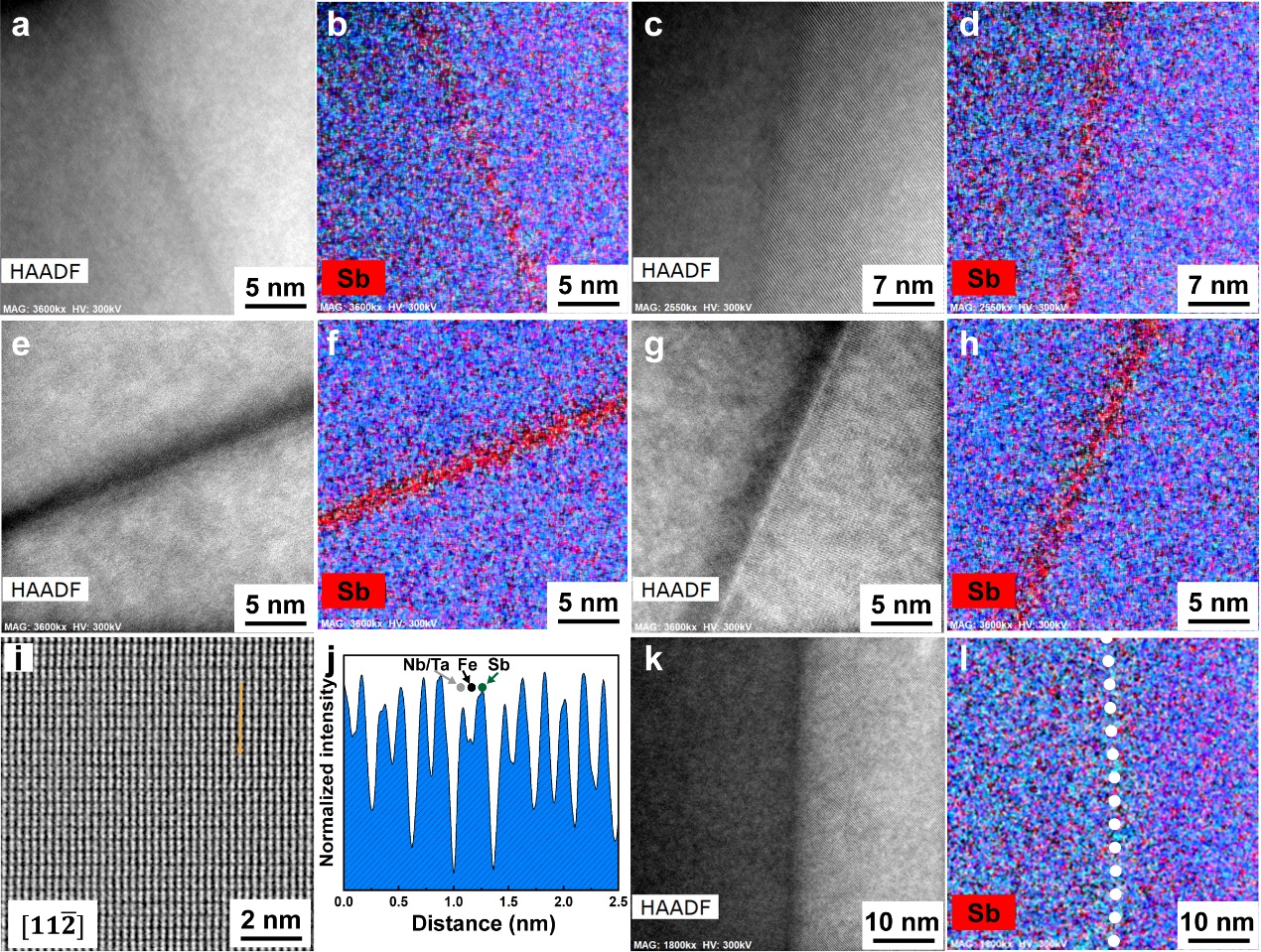


**Fig. S4 TEM and EDS mapping images of the Nb_0.55_Ta_0.40_Ti_0.05_FeSb samples with different annealing process.** **a-d,** The grain boundary of as-hot-pressed sample. **e-j,** The TEM images and EDS mapping (e-h) and the STEM and line scan (i,j) of the sample after conventional vacuum annealing at 1123 K for 6 days. **k,l,** The sample after Sb-pressure control annealing at 1143 K for 2 days, where the Sb-rich region at grain boundary is eliminated.


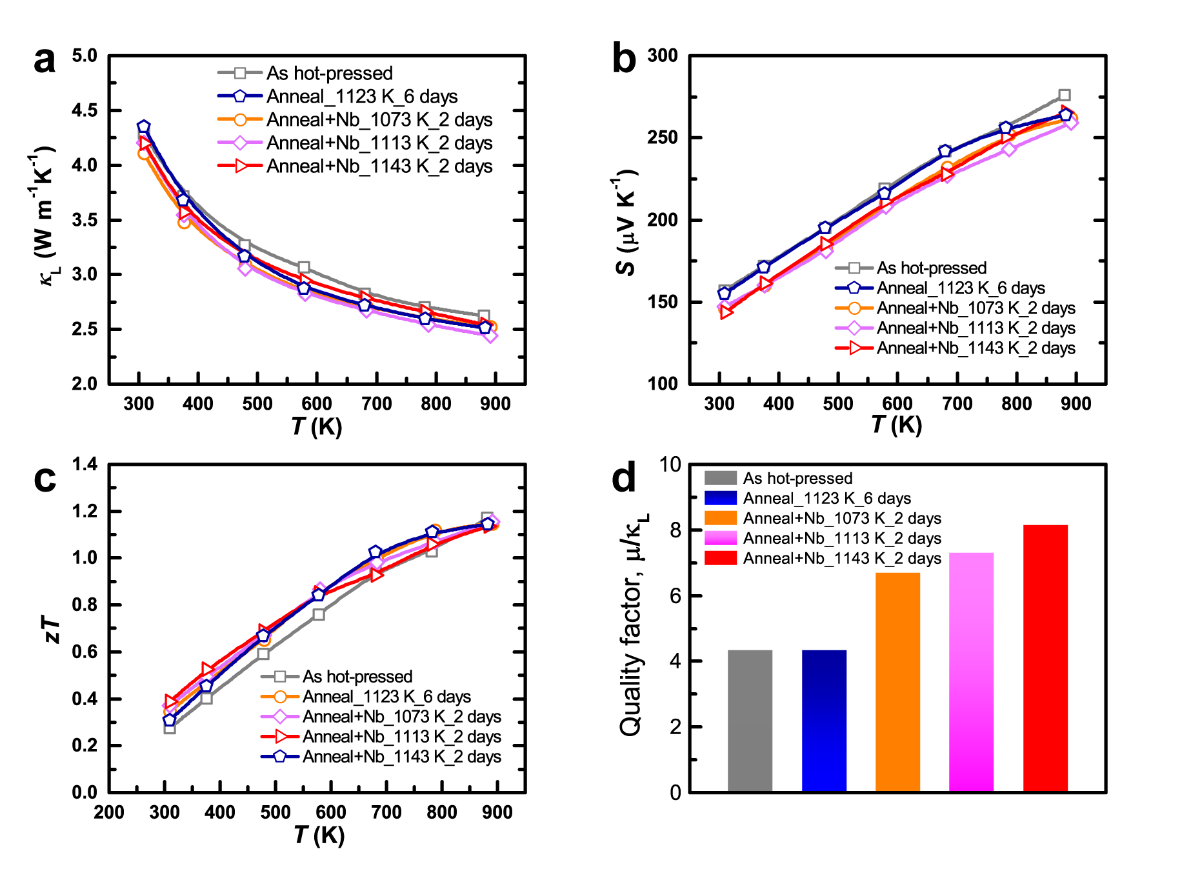


**Fig. S5 The thermoelectric transport properties of Nb_0.55_Ta_0.40_Ti_0.05_FeSb samples under different annealing condition.** **a,** The temperature dependent lattice thermal conductivity (*κ_L_*). **b,** The Seebeck coefficient (*S*). **c,** The *zT* value. **d,** Quality factor near room temperature.


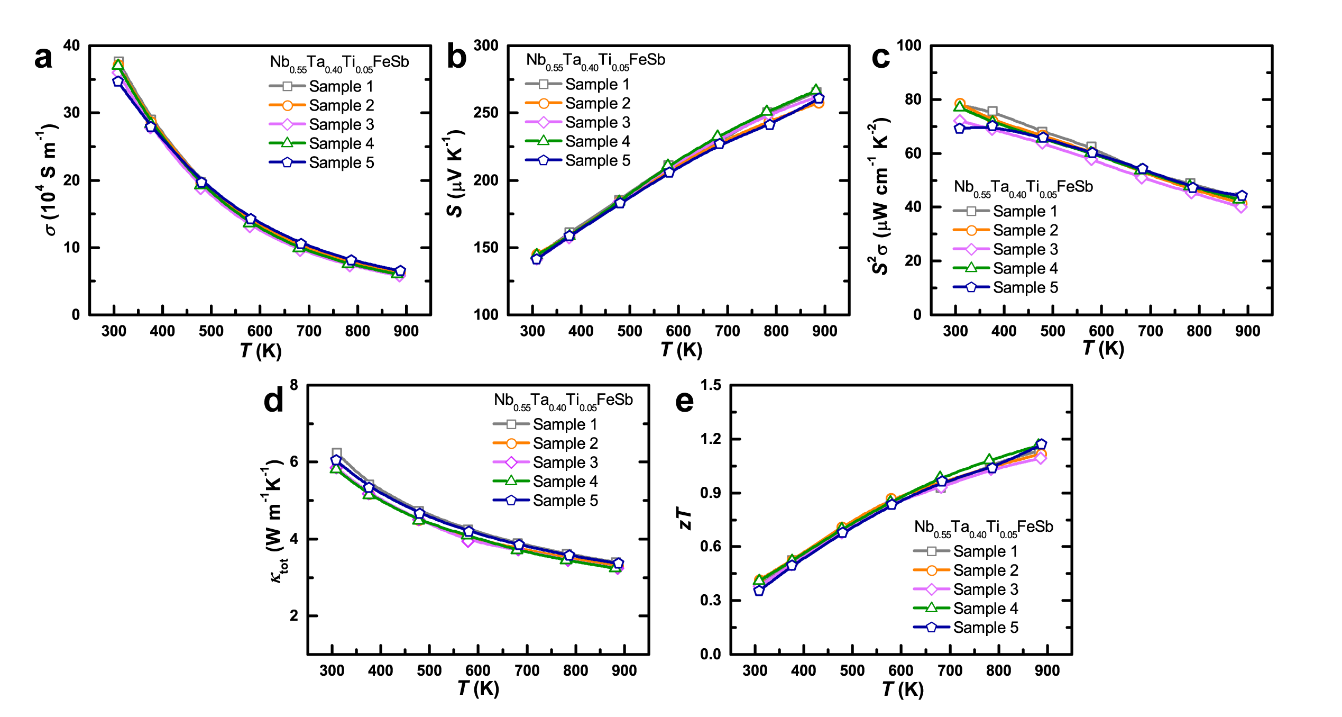


**Fig. S6 The** **reproducibility of the thermoelectric performance of the Nb_0.55_Ta_0.40_Ti_0.05_FeSb.** **a,** The temperature dependent electrical conductivity (*σ*). **b,** Seebeck coefficient (*S*). **c,** Power factor (*S^2^σ*). **d,** Total thermal conductivity (*κ_tot_*). **e,** *zT* value.


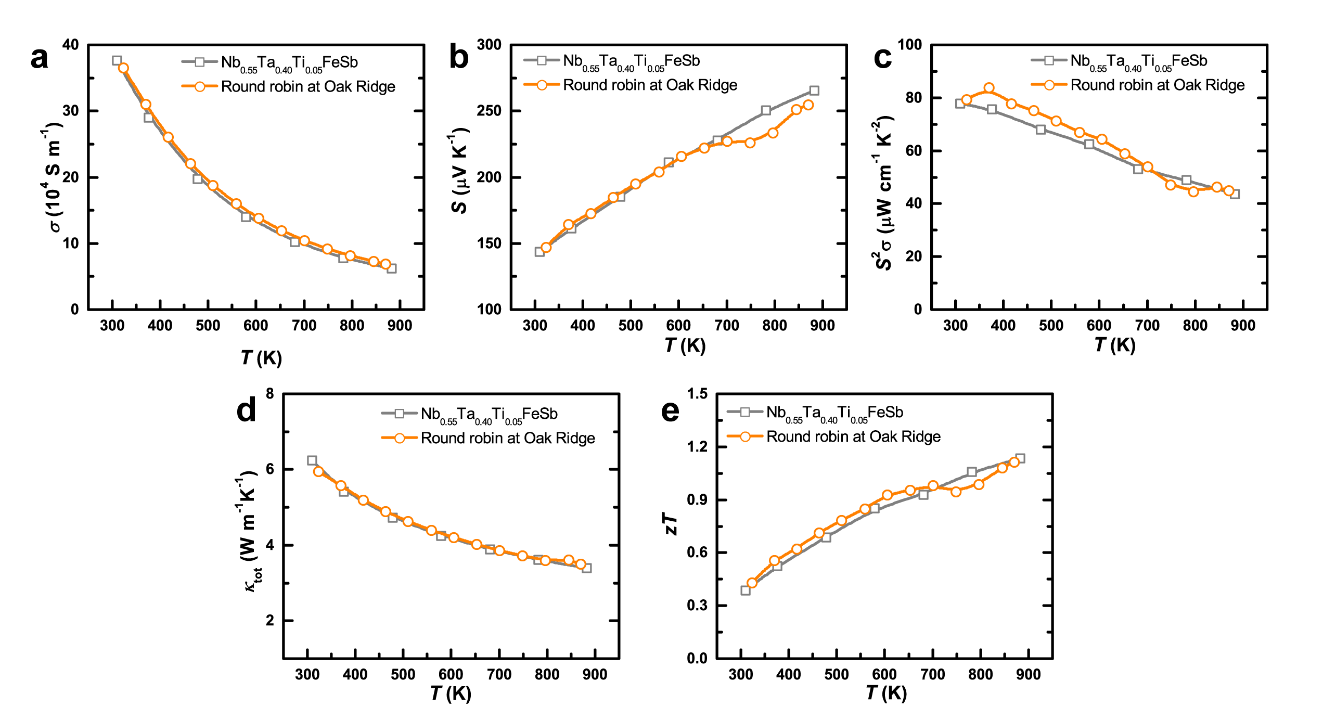


**Fig. S7 The round robin test of Nb_0.55_Ta_0.40_Ti_0.05_FeSb conducted at Oak Ridge national laboratory.** **a,** The temperature dependent electrical conductivity (*σ*). **b,** Seebeck coefficient (*S*). **c,** Power factor (*S^2^σ*). **d,** Total thermal conductivity (*κ_tot_*). **e,** *zT* value.

**
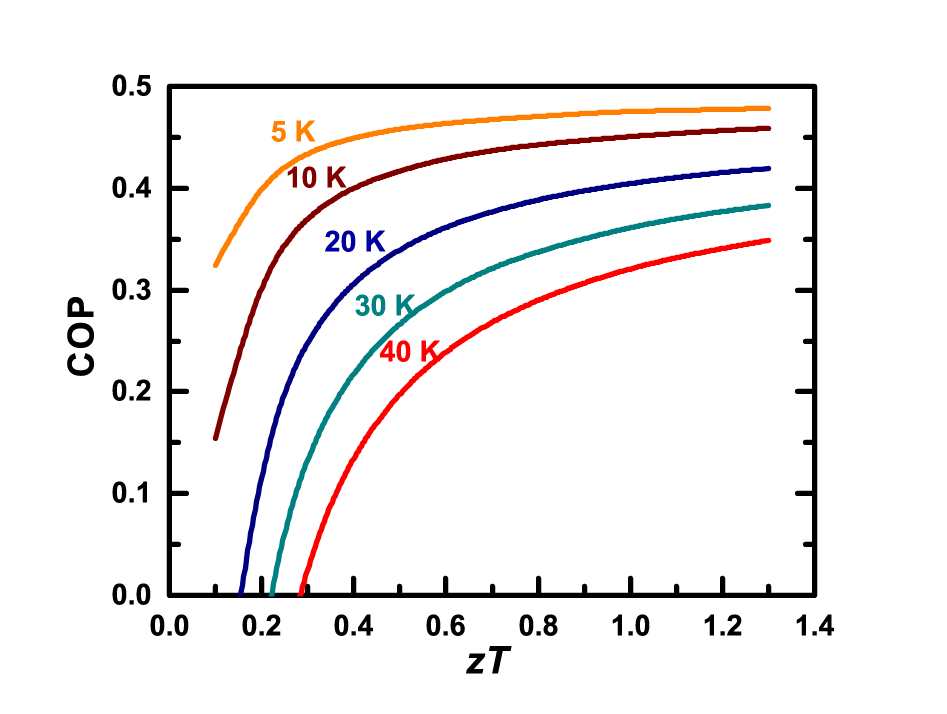
**

**Fig. S8** **The COP at the maximum cooling density with different zT and Δ*T*.**


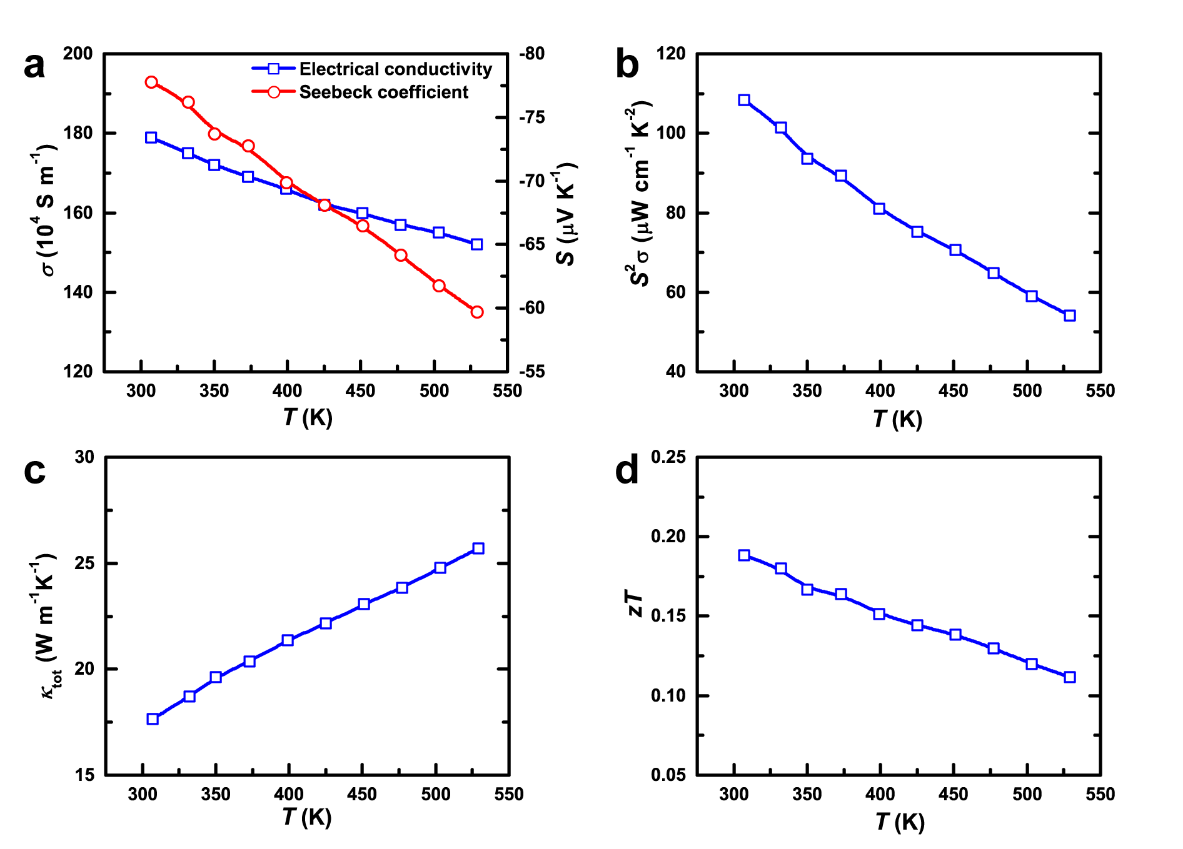


**Fig. S9 The thermoelectric properties of YbAl_3_ sample used in this work.** **a,** The temperature dependent electrical conductivity (*σ*) and Seebeck coefficient (*S*). **b,** Power factor (*S^2^σ*). **c,** Total thermal conductivity (*κ_tot_*). **d,** *zT* value.


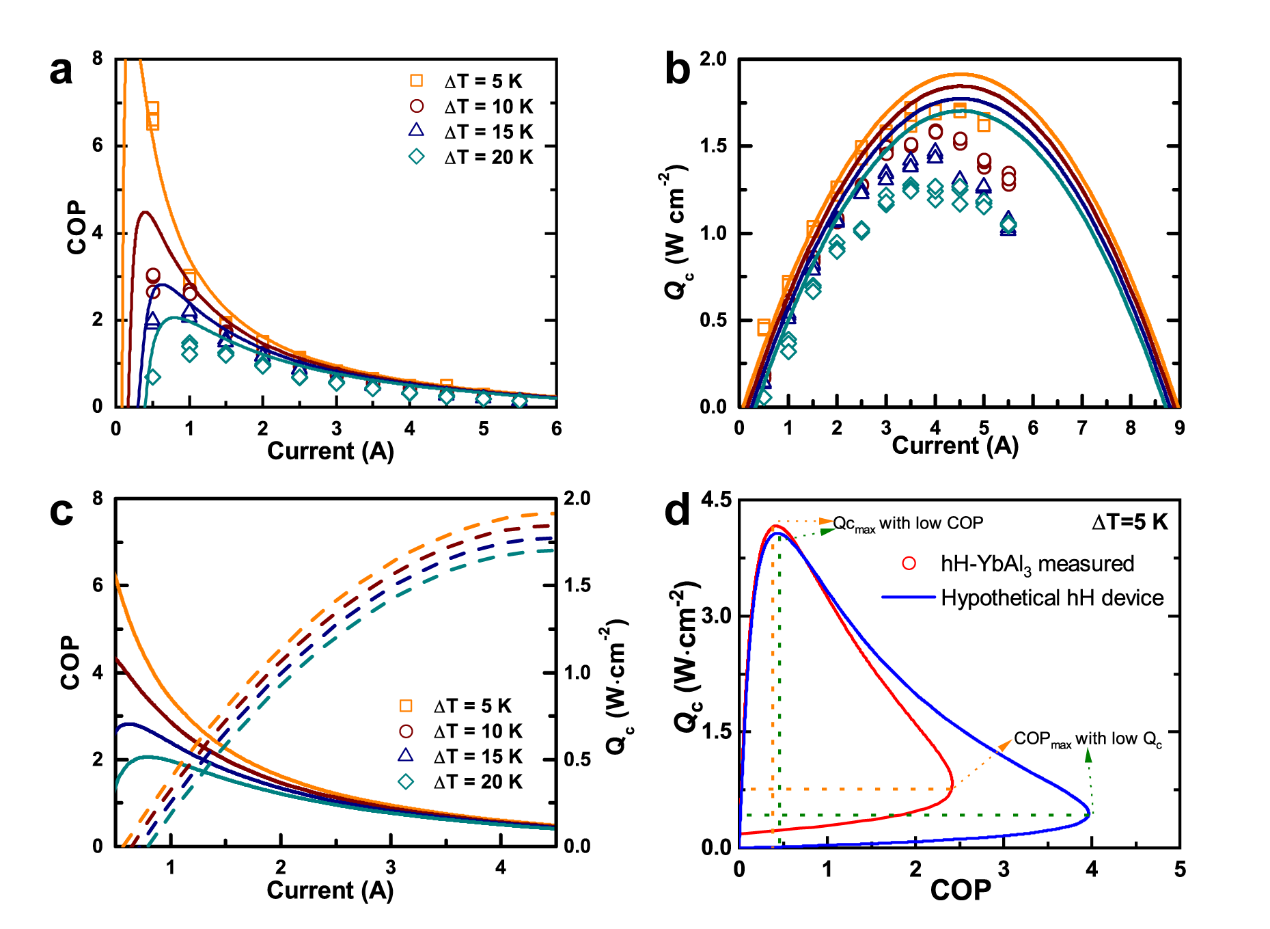


**Fig. S10 The cooling properties of the (Bi,Sb)_2_(Te,Se)_3_-based and hH-based devices.** **a,** Current dependent COP of (Bi,Sb)_2_(Te,Se)_3_-based device. **b,** Cooling heat flow (*Q_c_*) of of (Bi,Sb)_2_(Te,Se)_3_-based device. The BiTe materials are p-type Bi_0.33_Sb_1.67_Te_3_ and n-type Bi_2_Te_2.7_Se_0.3_. **c,** Current dependent COP and *Q_c_* of (Bi,Sb)_2_(Te,Se)_3_-based device. **d,** COP dependent *Q_c_* of hH-based device. The orange and olive lines indicate the high *Qc_max_* with low COP and high COP with low *Q_c_* conditions.


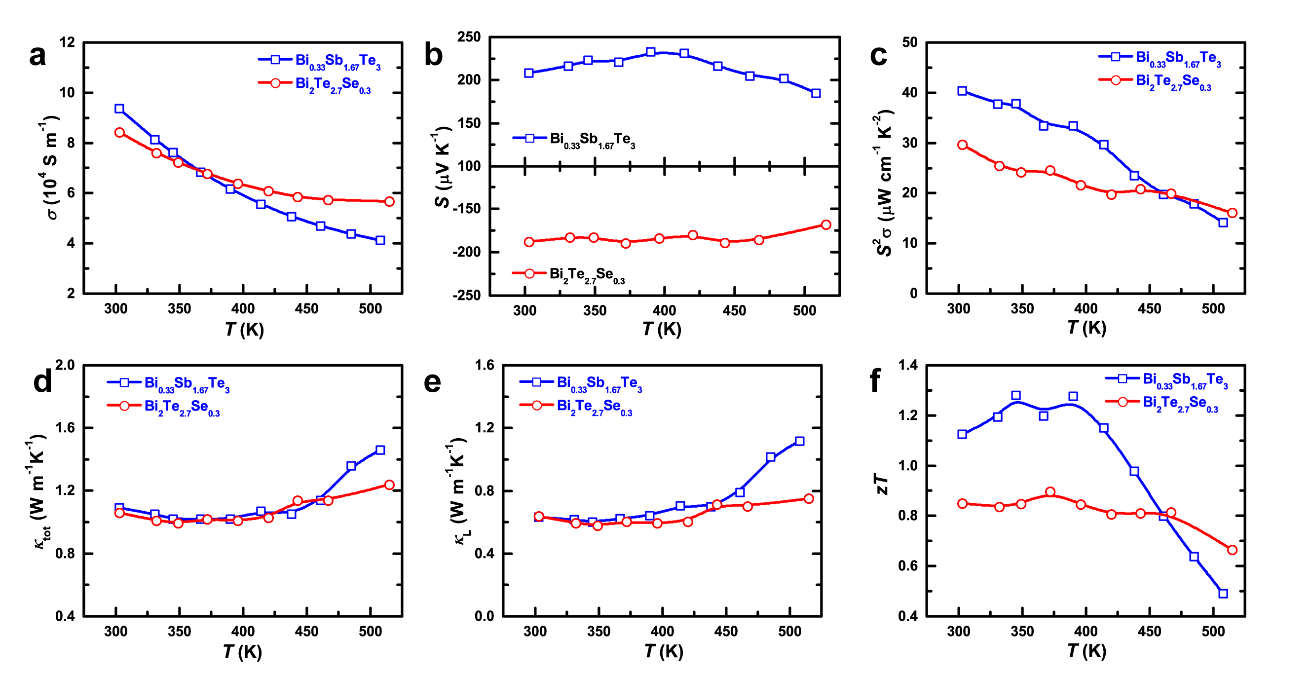


**Fig. S11 The** **thermoelectric transport properties of (Bi,Sb)_2_(Te,Se)_3_-based samples used in this work.** **a,** The temperature dependent electrical conductivity (*σ*). **b,** Seebeck coefficient (*S*). **c,** Power factor (*S^2^σ*). **d,** Total thermal conductivity (*κ_tot_*). **e,** Lattice thermal conductivity (*κ_L_*). **f,** *zT* value. The BiTe materials are p-type Bi_0.33_Sb_1.67_Te_3_ and n-type Bi_2_Te_2.7_Se_0.3_.


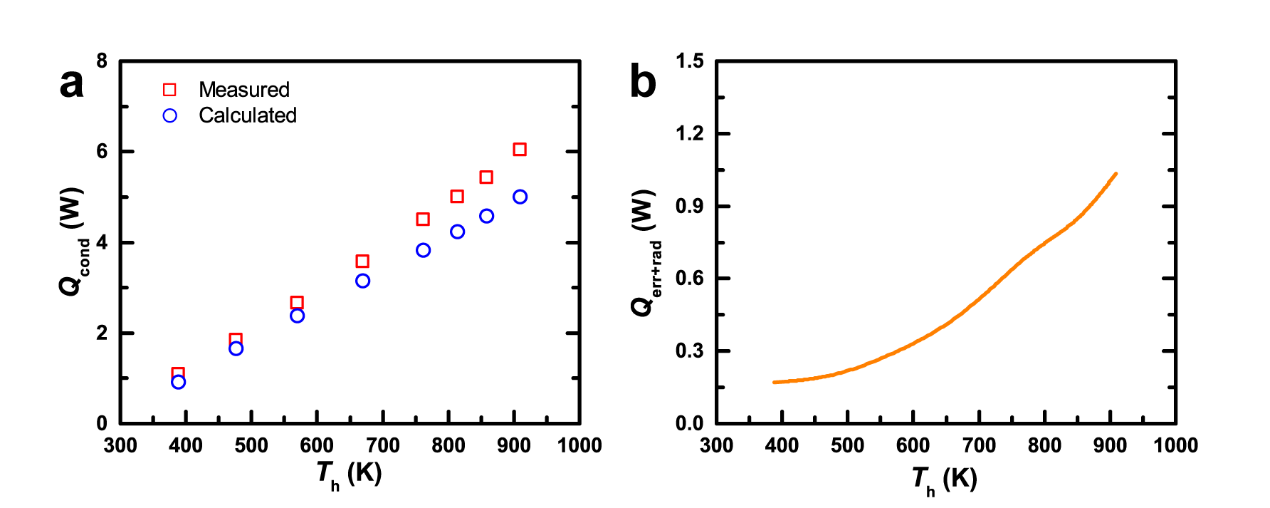


**Fig. S12 The calibration of thermal radiation and heat flow measurement.** **a,** The comparison of measured and theoretical heat flow of the dummy device, which is made of two legs of the standard Zr_0.44_Hf_0.44_Ti_0.12_NiSn_0.9_Sb_0.01_ materials. **b,** The temperature dependent thermal radiation and error of this measurement^1^.


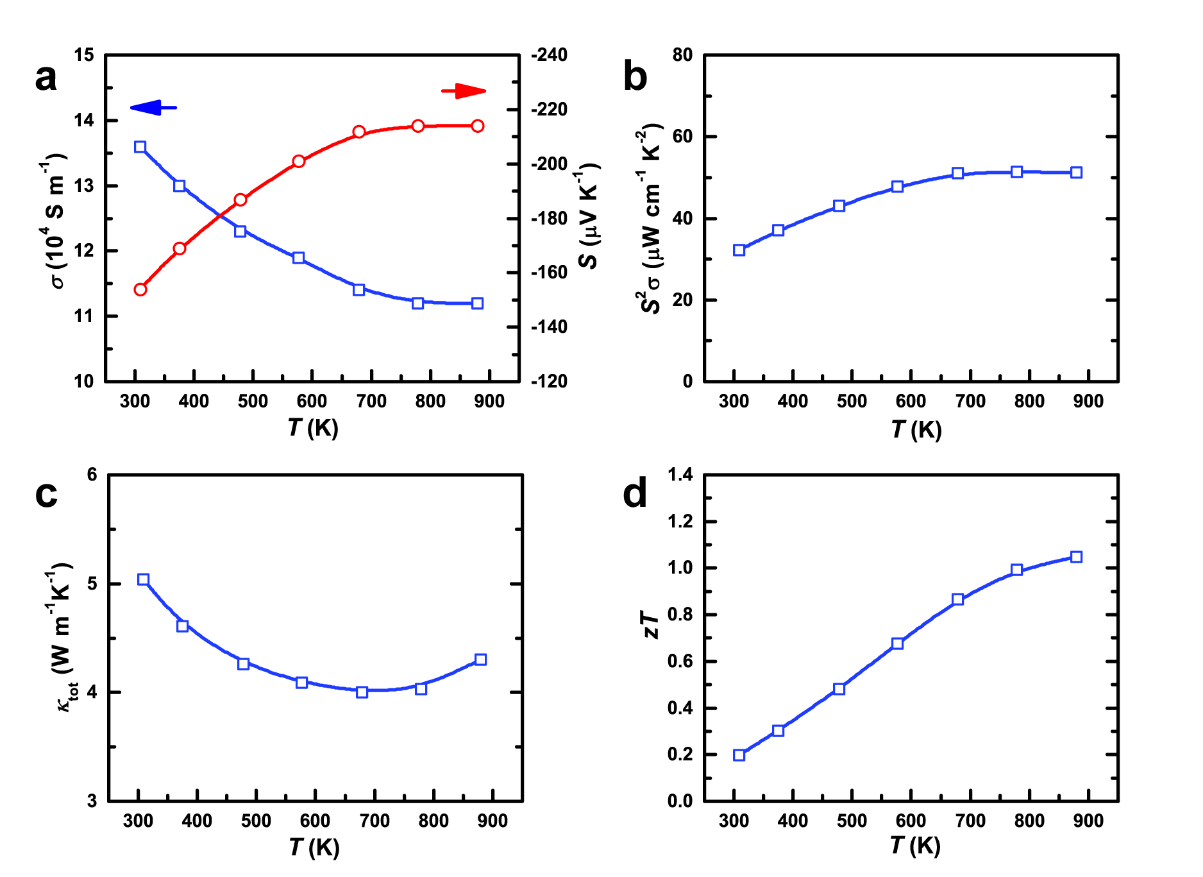


**Fig. S13 The thermoelectric transport properties of** **Zr_0.44_Hf_0.44_Ti_0.12_NiSn_0.9_Sb_0.01_ sample prepared in this work.** **a,** The temperature dependent electrical conductivity (*σ*) and Seebeck coefficient (*S*). **b,** *PF*. **c,** total thermal conductivity (*κ_tot_*). **d,** zT value.





**Fig. S14 The contact resistance of YbAl_3_ n-type leg, measured by a homemade four-probe device.**

**Table S1** Commercial high cooling power Bi_2_Te_3_-based micro-TECs.

| **Company** | **Model** | **I_max_ (A)** | **V_max_ (V)** | **ΔT_max_ (K)** | **Q_cmax_ (W)** | **Height (mm)** | **Leg Length (mm)** | **Maximum Cooling Density (W cm^-2^)** |
| --- | --- | --- | --- | --- | --- | --- | --- | --- |
| **Ferrotec^2^** | 20034/008/009AN | 0.9 | 1.2 | 65 | 0.6 | 0.9 |  | 14 |
| **Kelk^3^** | KSGH018 | 1.5 | 2.2 | 76 | 1.8 | 0.7 | ~0.2 | 45 |
| **Thermion^4^** | 1TMC04‐072‐03 | 5.85 | 8.3 | 70 | 28.6 | 1 | 0.3 | 21.3 |
| **Marlow^5^** | MT09-0.8A-01AN | 0.8 | 1.1 | 69 | 0.6 | 1.05 |  | 11 |

**Table S2** The parameters of p-type hH and BiTe materials near room temperature used in this study for high cooling density ($Q_{c_{max}}$) consideration.

| **Parameters** | **p-type Nb_0.55_Ta_0.40_Ti_0.05_FeSb** | **p-type Bi_0.33_Sb_1.67_Te_3_** |
| --- | --- | --- |
| ***T_c_* (K)** | 298 | 298 |
| ***∆T* (K)** | 5 | 5 |
| ***z* (K^-1^)** | 1.25e-3 | 3.6e-3 |
| ***PF* (μW·cm^-1^·K^-2^)** | 78 | 40 |
| ***κ* (W·m^-1^·K^-1^)** | 6.24 | 1.09 |
| ***l* (mm)** | 7.7 | 7.7 |

**Supplementary Note 1. Numerical analysis of TE device^6,7^:**

The TE legs are subdivided along the current direction into n elements, assuming that the temperature *T_i_* is constant in each segment. Thus, we will have constant TE properties in each segment, including thermal conductivity *κ*(*T_i_*), resistivity *𝜌*(*T_i_*), and Seebeck *S*(*T_i_*). The heat flow from the left side into the segment is:

${Q(i)}_{in}=\kappa(T_{i})A_{p}(T_{i}-T_{i+1})/dx$, (S.1)

Where *A_p_* is the cross-section area of the leg, dx is the length of the segment, *T_i_* and *T_i_*_+1_ are the temperature of left and right side of the segment, respectively.

The heat flow from the right side into the segment is:

${Q(i)}_{out}=\kappa(T_{i})A_{p}(T_{i+1}-T_{i+2})/dx$, (S.2)

The joule heat generated by the current is:

${Q(i)}_{joule}=I^{2}\rho(T_{i})dx/A_{p}$, (S.3)

The Thomson heat can be calculated as:

${Q(i)}_{Thomson}=-IT_{i}(S_{i+1}-S_{i})$, (S.4)

The thermal radiation emitted from the segment surface to the environment:

${Q(i)}_{radiation}=-\varepsilon\sigma(T_{i}^{4}-T_{r}^{4})wdx$, (S.5)

where 𝜀 is the emissivity of the TE material, *𝜎* is the Stefan-Boltzmann constant, *T_r_* is the environment temperature, *w* is the perimeter of the cross-section area.

The following formula can be derived from energy balance in steady state:

${Q(i)}_{out}={Q(i)}_{in}+{Q(i)}_{joule}+{Q(i)}_{Thomson}+{Q(i)}_{radiation}$, (S.6)

Though solving this finite element problem, we will get the temperature distribution function *T_i_* along the leg, which enable us to obtain the basic parameters and performance of the device. The total heat flow transfer into the legs is:

$Q_{in}=\kappa(T_{1})A_{p}(T_{1}-T_{2})/dx+T_{1}{IS}_{1}-\frac{1}{2}\left( I^{2}\rho(T_{1})dx/A_{p}-IT_{1}(S_{2}-S_{1})-\varepsilon\sigma(T_{1}^{4}-T_{r}^{4})wdx \right)$, (S.7)

Open circuit voltage of the device:

$V_{oc}=\sum_{1}^{n} S_{i}(T_{i}-T_{i+1})$, (S.8)

Dependence of voltage on current:

$V=V_{oc}-I\sum_{1}^{n} \rho(T_{i})dx/A_{p}$, (S.9)

The output power of the device:

$P_{out}=IV$, (S.10)

The energy conversion efficiency of device:

$\eta={P_{out}}/{Q_{in}}$, (S.11)

The COP of cooling device:

$\eta={Q_{in}}/{P_{out}}$, (S.12)

**Supplementary Note 2. Grain growth kinetics of hH under Sb pressure-controlled annealing:**

Fig. S15 shows the estimated grain growth kinetics and grain size as a function of annealing time under different temperatures for Nb_0.55_Ta_0.40_Ti_0.05_FeSb sample under Sb pressure-controlled annealing condition. As the grain growth is thermally activated, the rate of boundary motion is determined by annealing temperature (*T)*, the activation energy of boundary motion *(Q*_gb_), and driving force of grain growth (*p*). According to the Turnbull expression, the grain-boundary velocity (*x*) can be given as^8^:

$x=nmp=nm_{0}\exp\left( -\frac{Q_{\mathrm{gb}}}{k_{B}T} \right)p$, (S.13)

where *n* is the normal of grain-boundary segment, and *m* and *m_0_* are the actual and initial mobility of grain boundary, respectively. The *Q_gb_* can be reduced by eliminating any segregations at grain boundaries, for example, removing the Sb-rich phase in this study.

The *p* is the summation of acting forces for boundary migration:

$p=p_{\mathrm{gb}}-p_{p}-p_{s}$, (S.14)

The *p*_gb_ is the driving force determined by the surface energy of grain boundaries (*γ*) with average diameter of grains (*D*)^9^, which is the major source of *p*:

$p_{\mathrm{gb}}=2\gamma/D$, (S.15)

The *p_p_* is the retarding force due to the pinning force of the particles at grain boundaries determined by volume fraction (*V*_p_) and diameter of the particles (*r*). The elimination of Sb-rich phase at 1143 K can significantly reduce the *V*_p_ so that the *p_p_* is reduced.

$p_{p}=2V_{p}/r$, (S.16)

The *p*_s_ is the retarding force from solute drag effect determined by the interaction energy (*E*) and composition profile (*C*)^10^:

$p_{s}=N_{V}\int_{-\infty}^{+\infty} \left( C-C_{0} \right)\frac{dE}{dx}dx$, (S.17)

where *N*_v_ is the number of solute atoms per unit volume. In conventional annealing process, the solute atoms tend to segregate at the grain boundaries and thereby retard the migration of grain boundaries. However, during the Sb pressure-controlled annealing process, the elimination of Sb-rich phase also contributes to the reduction of *p*_s_. Overall, the grain growth of sample can be promoted significantly. The estimated *m* is 0.005, 0.127 and 4.700 cm^2^ V^-1^ s, at 1073 K, 1113 K and 1143 K, respectively. And the calculated activation energy *Q*_gb_ is ~8.2 eV for the hH alloys annealed with Sb-pressure control. Consequently, the estimated grain size of samples annealed at 1073 K, 1113 K and 1143 K for 2 days increased to ~5 μm, 25 μm and 150 μm, respectively, which is generally consistent with the experimental results (Fig. S15).


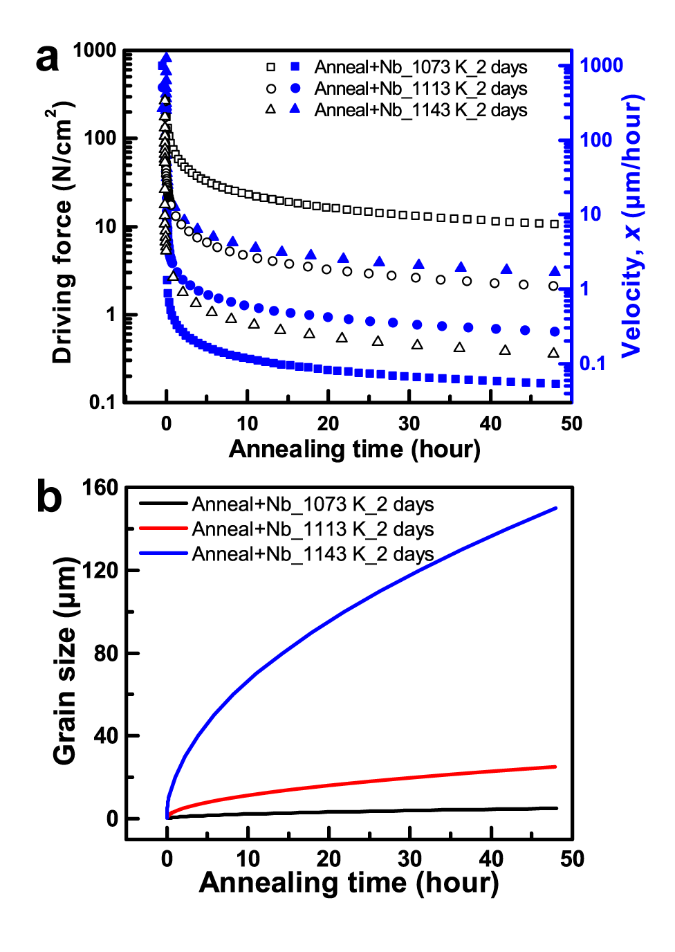


**Fig. S15** **Grain growth kinetics and grain size as a function of annealing time under different temperatures for Nb_0.55_Ta_0.40_Ti_0.05_FeSb sample under Sb pressure-controlled condition. a,** Driving force and velocity of grain growth. **b,** Grain size.

**Supplementary Note 3. The COP of cooling device with basic TE parameters：**

The COP value of the thermoelectric cooling device is determined by:

$COP=\frac{{ST}_{c}I-K\Delta T-RI^{2}/2}{S\Delta TI+RI^{2}}$, (S.18)

where *T_c_*, *ΔT*, *I*, *S*, *K* and *R* are the cold-side temperature, temperature difference, current, Seebeck coefficient, thermal conductance and the resistance of TE leg, respectively. Two most typical COPs to evaluate the performance of the TE cooling device are the COP of the maximum cooling density (COP_Q_) and the COP under zero temperature difference (COP_0_). The COP_Q_ decreases with increase of Δ*T* and *z* value, which is given by:

${COP}_{Q}=\frac{T_{c}}{2T_{h}}-\frac{\Delta T}{zT_{h}T_{c}}$, (S.19)

Theoretically, when Δ*T* is zero, COP_Q_ is pinned at 0.5 which is obviously independent with thermoelectric properties of material. This why the material with high power factor demonstrated both high $Q_{c_{max}}$and COP in device under low Δ*T*.

The COP_0_ is given by:

${COP}_{0}=\frac{{ST}_{c}}{RI}-\frac{1}{2}$, (S.20)

It is obvious the electrical properties (*PF*=*S^2^*/*ρ*) are important for the COP_0_, and the COP_0_ decreases with increase of applied current. A more comprehensive discussion about COP can be referred to reference [11].

As shown in Fig. S9c,d, high COP and high cooling density are two terminals of cooling device, which means maximum COP and cooling density cannot be achieved simultaneously. We can clearly observe that the COP reaches its peak value with low cooling density, and then decreases with the increase of cooling density (Fig. S9d). For instance, under a high heat load (*Qc_max_* of ~4 W·cm^-2^), the COP decreases to ~0.5 for both YbAl_3_-hH measured device and hypothetical hH device; while, under a low heat load (*Qc_max_* of ~0.75 and 0.5 W·cm^-2^ for hH-YbAl_3_ measured device and hypothetical hH device, respectively), the COP increases to ~2.5 and 4 for hH-YbAl_3_ measured device and hypothetical hH device, respectively.

**Supplementary References**

1. Li W*, et al.* Bismuth Telluride/Half-Heusler Segmented Thermoelectric Unicouple Modules Provide 12% Conversion Efficiency. *Adv. Energy Mater.* **10**, 2001924 (2020).

2. <http://www.ferrotec.com.cn/products/productinfo/84.html>. Ferrotec (2022).

3. <https://www.kelk.co.jp/english/products/thermo.html>. Kelk (2022).

4. <https://thermion-company.com>. Thermion. (2022).

5. <https://ii-vi.com/>. Marlow. (2022).

6. Kraemer D*, et al.* High thermoelectric conversion efficiency of MgAgSb-based material with hot-pressed contacts. *Energy Environ. Sci.* **8**, 1299-1308 (2015).

7. Kim HS, Liu WS, Chen G, Chua CW, Ren ZF. Relationship between thermoelectric figure of merit and energy conversion efficiency. *Proc. Natl. Acad. Sci. U.S.A.* **112**, 8205-8210 (2015).

8. Raabe D. 23 - Recovery and Recrystallization: Phenomena, Physics, Models, Simulation. In: *Physical Metallurgy (Fifth Edition)* (eds Laughlin DE, Hono K). Elsevier (2014).

9. Rios PR, Siciliano Jr F, Sandim HRZ, Plaut RL, Padilha AF. Nucleation and growth during recrystallization. *Materials Research* **8**, 225-238 (2005).

10. Cahn JW. The impurity-drag effect in grain boundary motion. *Acta Metall.* **10**, 789-798 (1962).

11. Zebarjadi M. Electronic cooling using thermoelectric devices. *Appl. Phys. Lett.* **106**, 203506 (2015).
